# Maternal and Early-Life Nutrition and Health

**DOI:** 10.3390/ijerph17217982

**Published:** 2020-10-30

**Authors:** Li-Tung Huang

**Affiliations:** 1 Department of Pediatrics, Kaohsiung Chang Gung Memorial Hospital, Kaohsiung 833, Taiwan; litung.huang@gmail.com; 2 Department of Medicine, Chang Gung University, Linkou 333, Taiwan

**Keywords:** maternal nutrition, prenatal nutrition, lactation, breastfeeding

## Abstract

Nutritional challenges prior to and during gestation, lactation, and early life are known to influence the lifelong health of the infant. In this editorial, I briefly discuss the 13 articles published in this Special Issue, “Maternal and Early-Life Nutrition and Health”. This Special Issue discusses topics including maternal nutrition behaviors, maternal overnutrition/obesity, maternal iron deficiency, breastfeeding, and others. This issue paves the way to better understand perinatal nutrition and how it can impact maternal and offspring health.

A total of 13 papers are published in this Special Issue entitled “Maternal and Early-Life Nutrition and Health”, and the topics include maternal nutrition behaviors [1,2,3,4] and maternal nutrition imbalances, such as maternal high-fat diets [5], maternal high-fructose diets [6], maternal obesity [7], and maternal iron deficiency [8]. Three articles investigated different factors in breastfeeding [9,10,11]. One article investigated the factors associated with preterm birth and low birth weight (LBW) among mothers of children in the United Arab Emirates [12]. Finally, Rossi et al. reported a false-positive diagnosis of methylmalonic aciduria in an infant due to maternal vitamin B12 deficiency [13].

Peacock et al. aimed to determine the effectiveness of antenatal intervention on health behaviors and pregnancy outcomes in women with obesity by accessing data from a large randomized controlled trial, the UK Pregnancies Better Eating and Activity trial. They suggested that antenatal interventions for women with obesity should stratify outcomes according to the severity of obesity. Their stratification protocol was as follows: Class I, II, and III (30–34.9, 35–39.9, ≥40 kg/m^2^, respectively). They demonstrated that participants with a BMI ≥ 40 kg/m^2^ had a significant decrease in gestational weight gain in response to antenatal intervention [1].

Barchitta et al. investigated the prevalence of folate deficiency and the determinants of dietary folate intake and folic acid supplementation among 397 pregnant Italian women. They hypothesized that folate deficiency leads to preterm birth and small size, according to gestational age. In this study, two thirds of women did not meet the current folate intake recommendations. The authors found high proportions of infants that were small relative to their gestational age, as well as infants who were large for their gestational age, among women with inadequate folate intake compared with those who met dietary recommendations. These findings highlight the need to improve the adherence of pregnant women to the recommended dietary folate intake and supplement use [2].

Ługowska and Kolanowski investigated the nutritional behavior of pregnant women in Poland. Their study enrolled 815 pregnant women in their first pregnancy. They found that the subjects ate an excessive amount of sweets and white bread and consumed insufficient quantities of fish, milk, and fermented milk drinks. They found that this inappropriate nutritional behavior was independent of education level. However, women with higher education had a slightly higher frequency of good nutritional practice. They concluded that the nutritional behavior of pregnant women as a whole needs improvement [3].

Postpartum Weight Retention (PWR) may increase the risk of adverse maternal health outcomes and also result in harmful health outcomes for the offspring. Nasreddine and colleagues studied the Mother and Infant Nutrition Assessment Cohort in Lebanon and Qatar [4]. They examined excessive PWR and its determinants at 6 months after delivery. They enrolled 183 pregnant women during their first trimester and followed up these mothers through pregnancy and after delivery. They found that Qatar women were associated with a risk of higher PWR compared to those living in Lebanon. The risk factors for higher PWR included excessive gestational weight gain and high dietary intakes of trans fat, sodium, cholesterol, and protein, which are usually consumed in Western diets. They concluded that dietary interventions spanning the entire period from pre-pregnancy through the postpartum period are needed to improve nutritional intakes of women of reproductive age in Lebanon and Qatar. The authors advocate dietary interventions may contribute towards the prevention of obesity in the offspring and hopefully decrease the obesity epidemic across generations [4].

Maternal obesity during pregnancy is now a public health burden. Tsai and colleagues have been working on a method to reprogram the adverse effects of maternal obesity. They administered a high-fat diet during lactation to pregnant rats and studied retroperitoneal adiposity in their adult male offspring. They found that maternal resveratrol administration reduced retroperitoneal adiposity and leptin dysregulation in rat offspring exposed to the maternal high-fat diet. Thus, resveratrol could ameliorate the negative effects of the maternal high-fat diet in adult male offspring [5]. 

In a rodent study, Liu and colleagues reported that maternal high-fructose diets impair the learning and memory performance of adult female offspring via an epigenetic mechanism of increased histone deacetylase 4 (HDAC4) activity in the hippocampus. They also demonstrated that maternal high-fructose diets during gestation and lactation also increase the hippocampal HDAC4 activity and suppress neurogenesis in adult female offspring. In addition, environmental enrichment was able to reverse the associated adverse effects by decreasing the hippocampal HDAC4 activity [6].

Hsu et al. wrote a review article regarding the programming effects of maternal obesity. They introduced five major underlying mechanisms of how maternal obesity exerts its effects on placental, fetus, and offspring development. They pointed out that the epigenome, gut–brain axis, inflammation, mitochondrial dysfunction, and brain-derived neurotrophic factor are among the five most investigated subjects in the field currently. In addition, they reviewed the current evidence of maternal resveratrol intake in human and animal studies of maternal obesity and considered resveratrol as a potential regimen in reprogramming adverse outcomes in the context of maternal obesity [7].

Hsieh et al. demonstrated that adult male rat offspring from maternal iron deficiency dam exhibited spatial deficit, decreased hippocampal BDNF mRNA expression and protein expression, and the enrichment of the *Bacteroidaceae* genus *Bacteroides* and *Lachnospiraceae* genus *Marvinbryantia*. They concluded that maternal iron deficiency leads to adult male rat offspring spatial deficit and is associated with alternations in the gastrointestinal microbiota and their metabolites. The findings of this study suggest that improving the nutritional status of pregnant women could have a positive effect on the future brain development of their offspring [8].

Breastfeeding is considered the best feeding practice for newborns due to its multiple health benefits. The mother’s self-efficacy of breastfeeding is related to the initiation and progression of the recommended minimum breastfeeding period. Santacruz-Salas et al. studied Spanish women’s expectations about exclusive breastfeeding and the effect of these expectations and other factors on breastfeeding during the first 6 months of motherhood. They conducted a prospective cohort study of 236 participants. They concluded that the achievement of exclusive breastfeeding may depend on a woman’s confidence in her ability to do so and on knowledge obtained through their social environment. Social environmental factors refer to a healthcare professional recommending other feeding options and/or the psychological pressure the woman may experience, including anxiety, doubts, problem-solving, and advice from others [9].

Scott et al. identified the prevalence and determinants of continued breastfeeding to 12 and 24 months in a study of 1450 Australian participants. They found that 31.8% of women breastfed for 12 months and 7.5% to 24 months. They concluded that the majority of Australian infants and their mothers are being deprived of the considerable benefits of continued breastfeeding. They emphasized that the majority of factors associated with the practice of continued breastfeeding were potentially modifiable [10].

Li and Binns presented a systematic review of studies on infection and breastfeeding in infants in Asia. They found that, when compared to the use of infant formula, breastfeeding is associated with significantly lower rates of diarrheal disease and lower respiratory tract infections, with a reduction of 50% or more, especially in the first six months of life [11].

Premature birth is defined as delivery before the start of the 37th week of pregnancy. LBW is characterized as a birth weight of less than 2500 g. Both preterm birth and LBW cause major public health problems worldwide. Taha et al. investigated the prevalence of preterm birth and LBW among mothers of children under two years of age in Abu Dhabi, United Arab Emirates. They found that the rates of preterm birth and LBW were high in their study region. The authors highlighted the need for interventions to tackle this public health issue, such as reducing the high rate of cesarean section delivery and improving maternal education [12].

Finally, Rossi et al. reported a false-positive diagnosis of methylmalonic aciduria in a newborn due to vitamin B12 deficiency originating from maternal malnutrition during pregnancy. Methylmalonic acidurias are a heterogeneous group of inborn errors of metabolism and, in this case, the error of propionate catabolism, which is characterized by gastrointestinal and neurometabolic manifestations. The authors suggest that monitoring maternal and offspring B12 levels, especially in the case of vegan and vegetarian mothers, will help to avoid the misdiagnosis of newborns with inborn errors of metabolism [13].

Nutritional programming has become a hot research topic. This Special Issue provides a broad view of nutritional challenges during early critical developmental periods and discusses their consequences, including socioeconomic impacts and disease occurrence. Future research in this field will improve pregnancy and offspring outcomes.
